# Standard hypothyroid treatment did not restore proper metabolic response to carbohydrate

**DOI:** 10.1007/s12020-020-02334-0

**Published:** 2020-05-13

**Authors:** Agnieszka Kozacz, Gilmara Gomes de Assis, Urszula Sanocka, Andrzej Wojciech Ziemba

**Affiliations:** 1 Department of Applied Physiology, Mossakowski Medical Research Centre, Polish Academy of Sciences, Pawinskiego 5 str., 02-106 Warsaw, Poland; 2 Endocrinology Outpatient Department, Masovian Hospital Bródno, Kondratowicza 8 str., 03-242 Warsaw, Poland

**Keywords:** Hypothyroid, Glucose tolerance, Energy expenditure, Thermogenesis, Noradrenaline

## Abstract

**Purpose:**

Hypothyroidism is associated with a lower metabolic rate, impaired glucose tolerance, and increased responsiveness of sympathetic nervous system to glucose ingestion. The Levothyroxine (LT4) monotherapy is the standard treatment for hypothyroidism; however to what extent this treatment restores the patients’ metabolism has not been verified. The aim of this study was to test the hypothesis that standard LT4 therapy may not restore proper metabolic response to carbohydrate ingestion.

**Methods:**

Energy expenditure, glucose tolerance, and catecholamine response to glucose ingestion were compared in 18 subjects with pharmacologically compensated hypothyroidism (PCH) and controls, at baseline and during oral glucose tolerance test conditions.

**Results:**

Metabolic rate was significantly lower in PCH (*P* < 0.0001). Glucose tolerance was decreased in this group with no differences in insulin resistance indicators between both groups. Adrenergic activity (*P* < 0.05) as well as adrenergic reaction to glucose ingestion (*P* < 0.001) were stronger in PCH.

**Conclusions:**

Standard treatment for hypothyroidism does not restore the normal metabolic reaction to carbohydrate which is observed in healthy people.

## Introduction

Hypothyroidism has an important impact on individuals’ glucose (Glu) tolerance, postprandial thermogenesis and sympathoadrenergic reactions to Glu ingestion [1, 2]. Due to a lack of specificity in the symptoms and signs, the current treatment for hypothyroidism is focussed on normalizing the levels of thyrotropin (TSH) and thyroid hormones (THs) by the administration of levothyroxine (LT4) [3], thus most of studies in such patients consist of comparing ‘on’ and ‘off’ conditions. Although the LT4 monotherapy treatment is standardly recommended by “Guidelines for the Treatment of Hypothyroidism” [3] not all patients are satisfied, some of them showing residual symptoms like psychological distress, thyroid symptoms, neurocognition, and general well-being impairment, depression, and anxiety [3–5]. Therefore, the metabolism of patients treated with LT4 may not necessarily correspond to healthy states, even in those with stabilized euthyroid conditions.

Glu tolerance is represented by standard reference ranges of plasma Glu. Normal Glu tolerance refers to the standardized values of fasting plasma Glu below 5.6 mmol/L and plasma Glu level below 7.8 mmol/L 2 h post Glu ingestion [6]. Thyroid dysfunction could be risk factor for Glu intolerance [7], in hypothyroidism, the Glu absorption is impaired, the peripheral Glu assimilation is delayed and gluconeogenesis is slower [8]. The available observations of changes in Glu metabolism during LT4 treatment are not consistent showing both alterations, as well as no differences in the levels of insulin (Ins) and Glu in fasting or post-Glu state after treatment [9–14] compared to healthy control.

The activity of sympathoadrenomedullary system, measured by catecholamine plasma concentrations, is much stronger in untreated hypothyroidism than in healthy individuals [2, 15–17]. Available observations of catecholamine levels in the plasma of patients during LT4 treatment relate to fasting values and are not consistent, documented both no differences between hypothroid and euthyroid groups at NA [18] and A [17], as well as higher NA concentration that decreases after LT4 therapy [15, 17].

THs play essential roles in thermogenesis [19], especially resting metabolic rate (RMR), a good measure of obligatory thermogenesis, is remarkably responsive to THs around the euthyroid state in humans [20]. Notwithstanding, some case study reported a lack of normalization of RMR during LT4 supplementation therapy in patients, despite of normalization of hormones levels [21, 22].

In our previous study we showed that THs play important role, also in postprandial thermogenesis, which refers to the additional energy expenditure associated with meal consumption [23]. In hypothyroid individuals, the postprandial thermogenesis is lower [2]. Unfortunately little is known about postprandial thermogenesis in a hypothyroid population [20].

Therefore, we decided to verify whether it is possible to restore metabolic responses to carbohydrate ingestion (i.e. Glu tolerance, postprandial thermogenesis, and sympathoadrenomedullary response) in hypothyroidism. Our hypothesis is that chronic treatment with L-T4, although normalizing the hormone levels may not properly restore metabolic responses to carbohydrate ingestion.

## Materials and methods

### Study population

The sample consisted of 18 patients (females, mean age 40.17 ± 3.06 years) with pharmacologically compensated hypothyroidism (PCH) caused by Hashimoto’s thyroiditis recruited from the Endocrinology Outpatient Department at the Masovian Hospital Bródno and 18 healthy controls matched by sex, age, body mass index (BMI) recruited by the announcement. General data is presented in Table 1. All subjects gave their written informed consent to be enrolled into this study, which was approved by the Local Ethics Committee of the Medical University of Warsaw. Inclusion criteria were undergoing L-T4-treated primary hypothyroidism for at least 3 years and showing compensated hypothyroidism—characterized by the maintenance of euthyreosis in peripheral blood. Exclusion criteria were taking drugs except LT4, metabolic disorders or others that affect basal energy expenditure (such as nervous system and musculoskeletal disorders or serious heart disease) and pregnancy.

**Table 1:** The general characteristics of the subjects

	Control group	PCH	*P* value
*n*	18	18	
Age (yr)	39.72 ± 2.85	40.17 ± 3.06	NS
BMI (kg/m^2^)	28.22 ± 1.37	28.51 ± 1.13	NS
TSH (mIU/L)	1.99 ± 0.22	2.16 ± 0.19	NS
fT3 (ng/L)	4.11 ± 0.07	3.95 ± 0.05	NS
fT4 (ng/L)	13.81 ± 0.66	14.58 ± 0.53	NS
fT3/fT4	0.23 ± 0.01	0.22 ± 0.01	NS
Triglycerides (mg/dL)	65.89 ± 7.14	66.61 ± 6.43	NS
Total cholesterol (mg/dL)	145.89 ± 6.48	144.61 ± 8.53	NS
High density lipoproteins cholesterol (mg/dL)	47.56 ± 3.18	46.10 ± 3.93	NS
Low density lipoproteins cholesterol (mg/dL)	88.84 ± 5.49	82.24 ± 6.70	NS

Values are disposed in means and standard error

*BMI* body mass index, *TSH* thyroid-stimulating hormone, *fT3* free triiodothyronine, *fT4* free thyroxine

### Study design

All tests were carried out between 7:00 and 12:00 a.m., at the room conditions of 22–24 °C and 40–50% humidity. The subjects attended to the laboratory following an overnight fast for the blood assessment. A catheter was inserted into the antecubital vein in one of subjects arm and allowing the resting in a supine position. Baseline/fasting blood samples were taken after 30 min of resting. Thereafter, the subjects were submitted to the 120-min oral Glu tolerance test (OGTT), in supine position, by drinking a solution containing 75 g of Glu dissolved in 200 ml of lukewarm water, with repeated samples of blood collected at before ingestion, and at 30th, 60th, 90th, and 120th min. Fasting Glu, Ins, TSH, free triiodothyronine (fT3), free thyroxine (fT4), total cholesterol, high-density lipoproteins cholesterol, low-density lipoproteins cholesterol, and triglycerides plasma concentrations were analyzed. Glu and Ins were also analyzed during the whole OGTT timepoints, as well as plasma adrenaline (A) and noradrenaline (NA) concentration’s peak from resting, minute 90th and 120th of OGTT as demonstrated by Mathias et al. [24]. OGTT was chosen as a protocol, also because it is a standardized model of carbohydrate meal commonly used in postprandial thermogenesis researches. Indirect calorimetry was used during 20 min before the Glu ingestion for RMR calculation. VO_2_ and VCO_2_ were recorded from the last 5 min of every quarter of hour of OGTT for postprandial energy expenditure calculation. Oxygen uptake (VO_2_) and carbon dioxide production (VCO_2_) were determined by Vmax29-Sensor Medics (CareFusion, San Diego, CA, USA) gas analyzer, with the accuracy of ±0.02% for O_2_ and ±0.02% for CO_2_. Subjects were laying in supine position during the whole test.

### Biochemical determinations

TSH, fT3, and fT4 were assayed by electrochemiluminescence immunoassay “ECLIA” from Roche Diagnostics GmbH (Mannheim, Germany) on cobas e 601 immunoassay analyzer. CV 3.3–7.2% for TSH, 2.0–3.4% for fT3, and 2.7–3.6% for fT4. Lipid profile was assayed by enzymatic colorimetric test on cobas c 502 analyzer: high-density lipoproteins cholesterol by HDLC3 test, total cholesterol by CHOL2 test, triglycerides by TRIGL test all from Roche Diagnostics GmbH (Mannheim, Germany). CV 0.9% for high-density lipoproteins cholesterol, 1.8–1.9% for triglycerides, and 1.4–1.6% for total cholesterol. Fraction of low-density lipoproteins cholesterol was calculated using the Friedewald [25] formula: low-density lipoproteins cholesterol = total cholesterol−high-density lipoproteins cholesterol–triglycerides/5 (mg/dL). Plasma Glu concentration was determined spectrophotometrically using Glu oxidase with a Glu test from BioMaxima S. A. (Lublin, Poland) (CV < 3.00%). Plasma Ins was assessed by immunoradiometric assay using an INS-IRMA Kit from DIAsource ImmunoAssays S.A. (Louvain-la-Neuve, Belgium) (CV 6.5–6.1%). Plasma A and NA levels were determined by radioimmunoassay with a reagent kit 2-CAT RIA from BioSource Europe S. A. (Nivelles, Belgium) with CV 5.6–6.1% for A and 10.1–6.1% for NA.

### Calculations

Areas under the curves were calculated using trapezoidal method. The indices of β-cell function were calculated: insulinogenic index (IGI)–[IGI = (Ins at 30th min–fasting Ins (mU/L))/(Glu at 30th–fasting Glu (mg/dL))], oral disposition index (oDI)–[oDI = IGI/fasting Ins (mU/L)], area under the Ins curve (Ins_auc_) and ratio of Ins_auc_ to area under the Glu curve (Glu_auc_) (Ins_auc_/Glu_auc_). Ins sensitivity was estimated in four ways: by fasting Ins level, the homeostasis model of Ins resistance (HOMA-IR)–[HOMA-IR = fasting Ins (mU/L) × fasting Glu (mmol/L)/22.5], the quantitative Ins sensitivity check index (QUICKI)–[QUICKI = 1/(log(fasting Ins (mU/L) + log(fasting Glu (mg/dL)))] and the Matsuda index–(ISI_(comp)_)–[ISI_(comp)_ = 10,000/SQRT (fasting Glu (mmol/L) × fasting Ins (mU/L) × mean Glu_(0-120)_ (mmol/L) × mean Ins_(0–120)_ (mU/L))]. The values of Ins resistance indices were assessed in relation to limiting values of indicators for Polish population [26]. Basal daily energy expenditure were calculated on the basis of RMR and Harris–Benedict formula [27]. Mean values of VO_2_ and VCO_2_ recorded during a 20 min gap before the Glu ingestion were used to calculate RMR (expressed in kJ/h/kg). The areas under the curves of postprandial energy expenditure were used to determinate postprandial thermogenesis (expressed in kJ).

### Statistical analysis

Data are presented as means with standard errors (±SE). Normality of variables was assessed by Shapiro–Wilk test. Student’s *t*-test or the Cochran and Cox test were used for the parametric data, depending on homogeneity of variance (assessed by Levene and Brown–Forsythe tests). Non-parametric data were compared by Mann and Whitney *U* test. Comparison of dependent variables of a given groups was calculated by dependent *t*-test for paired samples or the Wilcoxon signed-rank test. *P* < 0.05 was accepted as the level of significance. Statistica version 5 package was used (Statsoft Inc., Tulsa, OK, USA). Energy expenditure and blood Glu concentration were compared between groups by two-way analysis of variance (ANOVA) followed by multiple comparisons Newman–Keuls test. Plasma Ins, A, and NA were compared between groups by Mann and Whitney *U* test. Intra-group analysis of these variables were performed using the Wilcoxon signed-rank test.

## Results

### Energy expenditure

**Fig. 1:**
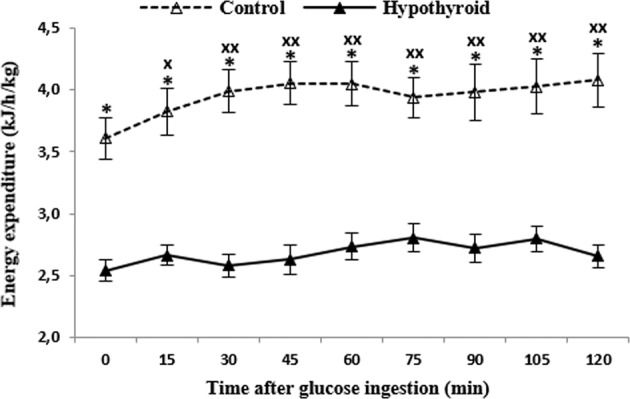
Changes in energy expenditure during 120 min oral glucose tolerance test in treated hypothyroid subjects (▴) and healthy control (▵). Values are disposed in means and standard error. “X” represents differences from fasting values (*P* < 0.05), “XX” (*P* < 0.01). “*” represents differences between groups (*P* < 0.001)

RMR and the energy expenditure throughout the test were significantly lower in PCH than in the controls (*P* < 0.0001). Two-way analysis of variance demonstrated a significant time factor (*P* < 0.001) and group factor (*P* < 0.0001) for energy expenditure. There was no time × group factors interaction. Post-hoc analysis revealed that energy expenditure was significantly lower (*P* < 0.0001) in PCH than in the controls at each time point (Fig. 1).

After Glu ingestion, the energy expenditure significantly increased already at minute 15th (*P* < 0.05) and remained elevated throughout OGTT (*P* < 0.01), but only in control group. Postprandial thermogenesis values in PCH was lower than in the controls (16.98 ± 6.29 vs. 47.29 ± 9.36 kJ) (*P* < 0.03).

In PCH, values of basal daily energy expenditure based on RMR and Harris–Benedict’s were 1144.67 ± 50.63 and 1547.89 ± 40.14 kcal/d, respectively. The measured RMR values were lower than the calculated by Harris–Benedict's (*P* < 0.0001). In the controls, Harris–Benedict’s were 1487.62 ± 45.83 kcal/d. There was no significant difference from the values obtained from RMR (1561.82 ± 126.21 kcal/d).

### Glu tolerance

**Fig. 2:**
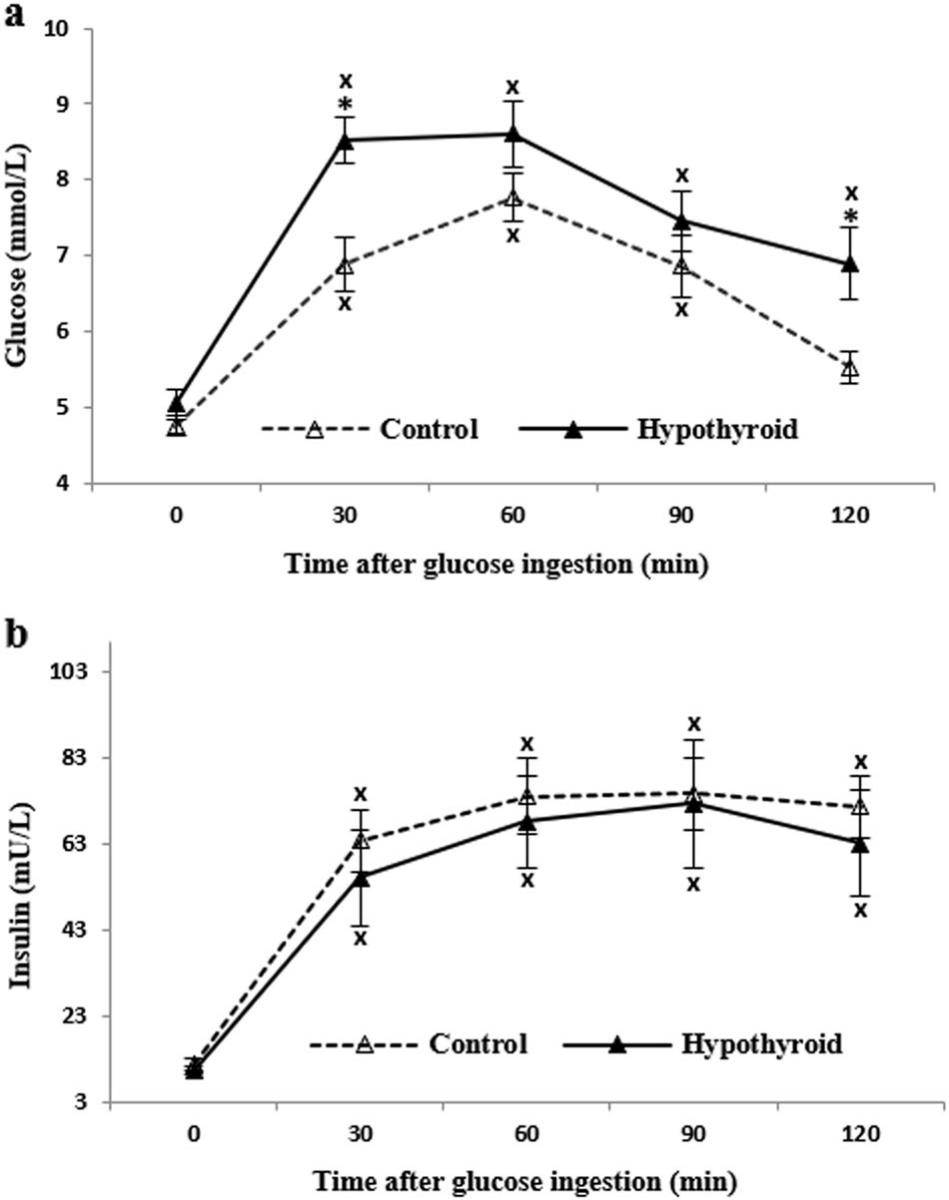
Plasma glucose and insulin concentrations at fasting state and during oral glucose tolerance test in treated hypothyroid subjects (▴) and healthy control (▵). Values are disposed in means and standard error. “X” represents differences from fasting values (*P* < 0.01), “*” represents differences between groups (*P* < 0.05)

Fasting Glu and Ins concentrations were not different between the groups. Two-way ANOVA showed a significant factor of time (*P* < 0.0001) and group (*P* < 0.05) for the mean values of Glu during OGTT (Fig. 2a). There was no time × group factors interaction. Post hoc analysis revealed that Glu values were significantly higher in PCH than in the controls at min 30th (*P* < 0.001) and 120th (*P* < 0.05). The Glu curve achieved the highest point at min 30th in PCH and min 60th in the controls. Maximal values of Glu were not different between groups. At min 120th, the Glu in PCH remained significantly elevated compared to baseline (*P* < 0.001). There was no significant difference between baseline and min 120th for the controls Glu (Fig. 2a). Glu_auc_ was significantly different (*P* < 0.05) (Table 2).

**Table 2:** Area under the curve of glucose (Glu_auc_), indices of β-cell function, and insulin resistance indicators of the subjects

		Control group	PCH	*P* value
Glu_auc_ (mmol/L min)		228.45 ± 22.49	311.74 ± 31.56	<0,05
Ins_auc_ (mU/L min)		6215.21 ± 599.00	5738.79 ± 1084.83	NS
Ins_auc_/Glu_auc_		31.50 ± 4.35	17.53 ± 2.99	<0.05
IGI		2.21 ± 0.64	0.79 ± 0.21	<0.05
oDI		0.22 ± 0.06	0.08 ± 0.02	<0.05
Fasting Ins (mIU/L)	(IR > 8.8)	11.84 ± 1.18	10.36 ± 0.88	NS
HOMA	(IR > 2.1)	2.52 ± 0.30	2.16 ± 0.17	NS
QUICKI	(IR < 0.34)	0.34 ± 0.01	0.34 ± 0.00	NS
ISI_(comp)_	(IR < 7.3)	4.32 ± 0.42	4.95 ± 0.65	NS

Ins_auc_—AUC of insulin, Ins_auc_/Glu_auc_—ratio of AUC of insulin to AUC of glucose, IGI—insulinogenic index, oDI—oral disposition index, HOMA—homeostasis model of insulin resistance, QUICKI—quantitative insulin sensitivity check index, ISI_comp_—Matsuda index, (IR…) presents insulin resistance limiting values of indicators for the Polish population [26]. Values are disposed in means and standard error.

No difference between PCH and the controls was identified in the Ins curve with maximal values achieved at min 90th in both groups and remained significantly elevated until min 120th (*P* < 0.01) (Fig. 2b). There was no difference in calculated IRI_auc_ between groups. IRI_auc_ to Glu_auc_ ratio (IRI_auc_/Glu_auc_) was significantly smaller (*P* < 0.05) in PCH when compared to the controls. IGI and oDI were significantly lower in PCH than in control group (*P* < 0.05). No difference were found in fasting Ins, HOMA, QUICKI, and ISI_(comp)_ between groups (Table 2).

### Plasma catecholamine pre–post Glu

**Fig. 3:**
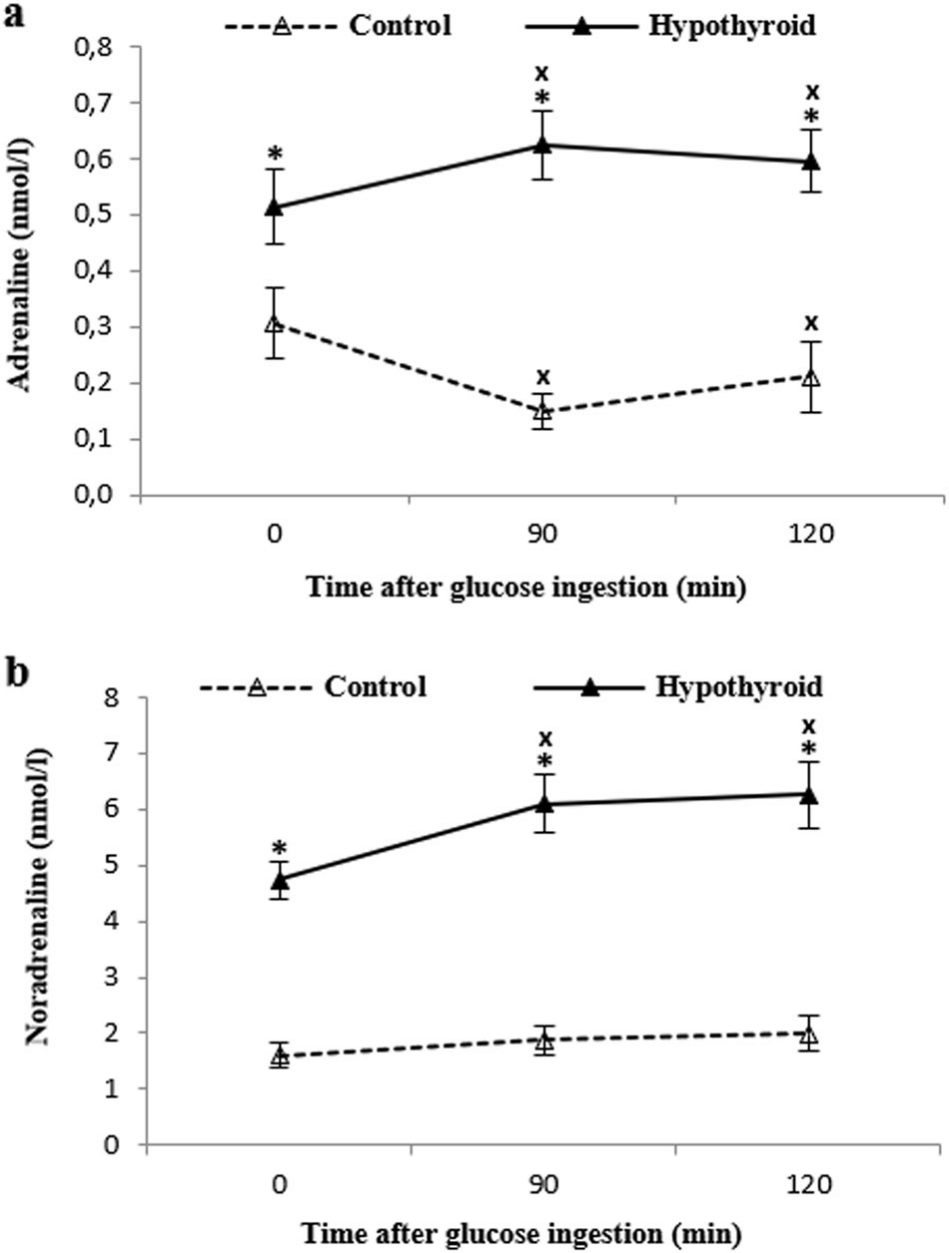
Plasma adrenaline and noradrenaline concentrations at fasting state and during oral glucose tolerance test in treated hypothyroid subjects (▴) and healthy control (▵). Values are disposed in means and standard error. “X” represents differences from fasting values (*P* < 0.05), “*” represents differences between groups (*P* < 0.05)

Plasma A concentrations were significantly increased in PCH than in the controls both at rest (*P* < 0.05), and at min 90th and 120th of OGTT (*P* < 0.001). At min 90th, plasma A concentrations were significantly decreased in the controls (*P* < 0.01) and increased in PCH (*P* < 0.05), compared to baseline. At min 120th, plasma A concentrations were still elevated in PCH and reduced in the controls (*P* < 0.05) (Fig. 3a).

Plasma NA concentrations were found to be significantly higher in PCH than in the controls in all time points (*P* < 0.001). At min 90th, there was a significant increase in plasma NA concentration in PCH (*P* < 0.01) which remained elevated at min 120th (*P* < 0.05). No changes were observed for the controls (Fig. 3b).

## Discussion

Based on results, it is possible to affirm that the metabolic response to carbohydrates is not properly restored in patients participating in our study, who are hypothyroid under L-T4 treatment. Disturbances in Glu tolerance can be manifested by the fasting and post-Glu ingestion levels [6]. In our study, both control and PCH group showed normal Glu tolerance. Also, the levels of plasma Glu and Ins in fasting state did not differ between groups, in coherence with previous studies [14, 28]. However, some studies have reported both lower Glu [13, 29] and higher Glu and Ins in PCH [30].

In the present study, although the fasting Glu did not differ from the controls, the plasma Glu levels in PCH were higher at the first stage and at the end of OGTT, producing a greater Glu_auc_. Taking into consideration, that there were no differences in insulin level at any measurement point, higher plasma Glu levels at the first stage of OGTT indicate a delay in Glu uptake. That is in line with the general metabolic slowdown in the PCH group and it was confirmed by still higher Glu levels in PCH than in the fasting and control conditions. Glu uptake delay with greater Glu_auc_ was also observed in untreated hypothyroid patients compared to the healthy controls matched by age and BMI in our previews study. Importantly, in the untreated patients Glu levels returned to basal values at the end of OGTT [2].

In this study, there was no difference in Ins resistance in both groups. According to the analyzed indicators (fasting Ins, HOMA, QUICKI, and ISI_(comp)_), both groups were found to be insulin-resistant. Since Ins resistance is strongly associated with high BMI [31] and that both of our groups were overweight, we believe that the Ins resistance, in this case, was more likely to be a reflection of their BMI than their hypothyroid condition [32]. According to the mechanism proposed by Diamond et al. [33], even in insulin resistance normoglycemia can be maintained by adjusting β-cells insulin secretion to the body’s sensitivity to insulin. When experiencing a reduction in insulin sensitivity of 80% due to one of many possible causes (puberty, pregnancy, infection, increased adiposity), an individual would be predicted to mount a five-fold greater insulin response [34]. Thus, even in insulin-resistance, as long as these cells are able to enhance Ins secretion, the Glu tolerance remains normal. Glu intolerance occurs when an Ins resistance can no longer be compensated by pancreas β-cells production of Ins. With time, the β-cells begin to fail and initially, the postprandial plasma glucose levels and subsequently, the fasting plasma glucose concentration begin to rise, leading to the onset of overt diabetes [35, 36]. β-cell function indicators: Ins_auc_/Glu_auc_, IGI, and oDI in PCH were lower than in controls as was the case observed in other studies [9]. Therefore, we believe that in PCH there is a gradual deterioration in β-cell function, which is manifested through a loss of pancreas Ins secretion compensatory capability, during ongoing hypothyroidism, despite LT4 therapy. This resulted in higher glucose level paralleled with lower insulin secretion after glucose consumption observed in PCH. We believe that physiological insulin resistance in our healthy controls was compensated by their β-cells insulin secretion at sufficient level to maintain normoglycemia, while in the PCH the capacity of β-cells insulin secretion began to be insufficient. Thus, we observed the early stage of glucose intolerance.

Compiling results from both studies, it is observable that the Glu tolerance decays in time with hypothyroidism. Moreover, long-term LT4 therapy does not restore normal Glu tolerance in PCH. Regular evaluation of Glu metabolism during treatment is not a recommendation, according to both ATA, ETA, and AACE guidelines, for these patients [3, 4]; and the Italian Association of Clinical Endocrinologists (AME) & Italian Association of Clinical Diabetologists (AMD) [37] endorse to repeat Glu metabolism evaluation (by OGTT) only once, after the restoration of normal thyroid function. We would suggest considering a periodically OGTT for PCH regardless of the stabilization of TH.

RMR was lower in PCH. TH play a key role in shaping the RMR which has been already used for diagnosis and titrations in hypothyroidism [38]. While lower RMR is a characteristic of hypothyroid state, patients undergoing treatment should demonstrate normalized RMR levels. Such normalization was reported by Wolf et al. (1996), however, TSH-suppressive doses were used for this [39]. Although, lack of RMR increases despite increasing plasma fT3 level, was also reported [22]. The normalized TH blood concentration with slower RMR suggests a state of “tissue hypothyreosis condition” characterized by a difference between plasma THs and THs concentration and/or activity inside cells which is assumable when considering the complexity of mechanisms governing the proper tissue response to TH stimulation [40, 41]. Such phenomenon has already been observed in NA dynamics in non-treated hypothyroid subjects [42]. Other possible explanation of lower RMR can be resulting from deficiencies in other than triiodothyronine and thyroxine active substances secreted by the thyroid gland and/or THs-active intermediate metabolites [3, 43]. Some of the active substances from thyroid gland are present in desiccated thyroid extract, which may be one of the explanations of increased satisfaction with the therapy of patients taking desiccated thyroid extract than patients taking LT4 as noticed by Peterson et al. [5].

Likewise, postprandial thermogenesis was lower in PCH than control group, corroborating with the slow RMR. There are not many studies reporting postprandial thermogenesis in PCH. Similar to our results, no significant changes in postprandial thermogensis was observed either in hypohyroid, hyperhyroid, or euthyroid state by Al-Adsani et al. [20] although they were not compared to healthy control group, so that, those authors stated that postprandial thermogenesis values obtained in PCH were lower comparing to healthy standards [44, 45].

Additionally, PCH sympathetic activity was higher than healthy control group. In the present study pre-and post Glu ingestion A and NA levels were higher in PCH than in the controls as it has been also observed in untreated hypothyroid patients [2, 16]. This indicates that the PCH adrenergic reaction is not restored to the observed in healthy people. The increased sympathetic activity in untreated hypothyroid subjects may be a compensatory mechanism to achieve an appropriate level of tissue response to stimulation, since β-adrenoceptors responsiveness in hypothyroidism is reduced [16, 46]. Moreover, the rise in the level of A is an opposite reaction to that observed in healthy subjects [47, 48]. Since A is the hormone that exerts a strong thermogenic effect, it could be a way to increase thermogenesis which is reduced in hypothyroidism. However, if so, in both this and earlier study [2] it was ineffective.

It should be reported that this study addressed only female individuals and they might present different luteal phases. However, despite the heterogeneity of the groups in terms of their luteal phases, the analysis of the measured indicators showed no presence of distinct subgroups.

Concluding, we believe that although the currently recommended treatment for hypothyroidism does compensate THs level in blood, they do not accomplish to fully restore euthyreosis.

## Data Availability

The datasets generated during the current study are available from the corresponding author on reasonable request.

## References

[1] Mullur R, Liu Y-Y, Brent GA (2014). Thyroid hormone regulation of metabolism. Physiol. Rev..

[2] A. Kozacz, P. Grunt, M. Steczkowska et al. Thermogenic effect of glucose in hypothyroid subjects. Int. J. Endocrinol. (2014). 10.1155/2014/308017 10.1155/2014/308017 PMC3966342 24711817

[3] Jonklaas J, Bianco AC, Bauer AJ (2014). Guidelines for the treatment of hypothyroidism: prepared by the American Thyroid Association task force on thyroid hormone replacement. Thyroid.

[4] Wiersinga WM, Duntas L, Fadeyev V (2012). 2012 ETA guidelines: the use of L-T4 + L-T3 in the treatment of hypothyroidism. Eur. Thyroid J..

[5] Peterson SJ, Cappola AR, Castro MR (2018). An online survey of hypothyroid patients demonstrates prominent dissatisfaction. Thyroid.

[6] American Diabetes Association (2018). Classification and diagnosis of diabetes: Standards of Medical Care in Diabetes—2018. Diabetes Care.

[7] Nishi M (2018). Diabetes mellitus and thyroid diseases. Diabetol. Int..

[8] Duntas L, Orgiazzi J, Brabant G (2011). The interface between thyroid and diabetes mellitus. Clin. Endocrinol..

[9] Handisurya A, Pacini G, Tura A (2008). Effects of T4 replacement therapy on glucose metabolism in subjects with subclinical (SH) and overt hypothyroidism (OH). Clin. Endocrinol..

[10] Anantarapu S, Vaikkakara S, Sachan A (2015). Effects of thyroid hormone replacement on glycated hemoglobin levels in non diabetic subjects with overt hypothyroidism. Arch. Endocrinol. Metab..

[11] Hays JH, Silverman E, Potter BB (1994). Normal gastric inhibitory polypeptide response to oral glucose in hypothyroidism. J. Endocrinol..

[12] Kung AWC, Lam KSL, Pun KK (1990). Circulating somatostatin after oral glucose in hypothyroidism. J. Endocrinol. Invest..

[13] Owecki M, Nikisch E, Sowiński J (2006). Hypothyroidism has no impact on insulin sensitivity assessed with HOMA-IR in totally thyroidectomized patients. Acta Clin. Belg..

[14] Nada AM (2013). Effect of treatment of overt hypothyroidism on insulin resistance. World J. Diabetes.

[15] Polikar R, Kennedy B, Ziegler M (1990). Plasma norepinephrine kinetics, dopamine-beta-hydroxylase, and chromogranin-A, in hypothyroid patients before and following replacement therapy. J. Clin. Endocrinol. Metab..

[16] Nedvidkova J, Haluzik M, Bartak V (2004). Changes of noradrenergic activity and lipolysis in the subcutaneous abdominal adipose tissue of hypo- and hyperthyroid patients: an in vivo microdialysis study. Ann. N. Y. Acad. Sci..

[17] Velardo A, Del Rio G, Zizzo G (1994). Plasma catecholamines after thyrotropin-releasing hormone administration in hypothyroid patients before and during therapy. Eur. J. Endocrinol..

[18] Pinkney JH, Goodrick SJ, Katz JR (2000). Thyroid and sympathetic influences on plasma leptin in hypothyroidism and hyperthyroidism. Int. J. Obes..

[19] Silva JE (2003). The thermogenic effect of thyroid hormone and its clinical implications. Ann. Intern. Med..

[20] Al-Adsani H, Hoffer L, Silva JE (1997). Resting energy expenditure is sensitive to small dose changes in patients on chronic thyroid hormone replacement 1. J. Clin. Endocrinol. Metab..

[21] van Santen HM, Schouten-Meeteren AY, Serlie M (2015). Effects of T3 treatment on brown adipose tissue and energy expenditure in a patient with craniopharyngioma and hypothalamic obesity. J. Pediatr. Endocrinol. Metab..

[22] F. Martucci, G. Manzoni, G. Lattuada et al. Overweight/obese women with primary acquired hypothyroidism in appropriate levothyroxine replacement therapy are characterized by impaired whole body energy metabolism. Endocr. Abstr. (2013). 10.1530/endoabs.32.P1004

[23] Tappy L (1996). Thermic effect of food and sympathetic nervous system activity in humans. Reprod. Nutr. Dev..

[24] Mathias CJ, da Costa DF, McIntosh CM (1989). Differential blood pressure and hormonal effects after glucose and xylose ingestion in chronic autonomic failure. Clin. Sci..

[25] Friedewald WT, Levy RI, Fredrickson DS (1972). Estimation of the concentration of low-density lipoprotein cholesterol in plasma, without use of the preparative ultracentrifuge. Clin. Chem..

[26] Szurkowska M, Szafraniec K, Gilis-Januszewska A (2005). Wkaźniki insulinooporności w badaniu populacyjnym i ich wartość predykcyjna w określeniu zespołu metabolicznego. Prz. Epidemiol..

[27] Harris AJ, Benedict FG (1918). A biometric study of human basal metabolism. Proc. Natl Acad. Sci. USA.

[28] Rochon C, Tauveron I, Dejax C (2003). Response of glucose disposal to hyperinsulinaemia in human hypothyroidism and hyperthyroidism. Clin. Sci..

[29] Stanická S, Vondra K, Pelikánová T (2005). Insulin sensitivity and counter-regulatory hormones in hypothyroidism and during thyroid hormone replacement therapy. Clin. Chem. Lab. Med..

[30] B UU, Mn S, Km S (2015). Effect of insulin resistance in assessing the clinical outcome of clinical and subclinical hypothyroid patients. J. Clin. Diagn. Res..

[31] G. Daryabor, D. Kabelitz, K. Kalantar. An update on immune dysregulation in obesity-related insulin resistance. Scand. J. Immunol. **89**, e12747. 10.1111/sji.12747. Epub 29 Jan 2019 (Review). (2018) 10.1111/sji.12747 30593678

[32] Maratou E, Hadjidakis DJ, Kollias A (2009). Studies of insulin resistance in patients with clinical and subclinical hypothyroidism. Eur. J. Endocrinol..

[33] Diamond MP, Thornton K, Connolly-Diamond M (1995). Reciprocal variations in insulin-stimulated glucose uptake and pancreatic insulin secretion in women with normal glucose tolerance. J. Soc. Gynecol. Investig..

[34] Bergman RN, Ader M, Huecking K (2002). Accurate assessment of β-cell function. Diabetes.

[35] DeFronzo RA (2009). From the triumvirate to the ominous octet: a new paradigm for the treatment of type 2 diabetes mellitus. Diabetes.

[36] Galgani JE, Ravussin E (2012). Postprandial whole-body glycolysis is similar in insulin-resistant and insulin-sensitive non-diabetic humans. Diabetologia.

[37] Guastamacchia E, Triggiani V, Aglialoro A (2015). Italian Association of Clinical Endocrinologists (AME) & Italian Association of Clinical Diabetologists (AMD) Position Statement: diabetes mellitus and thyroid disorders: recommendations for clinical practice. Endocrine.

[38] McAninch EA, Bianco AC (2016). The history and future of treatment of hypothyroidism. Ann. Intern. Med..

[39] Wolf M, Weigert A, Kreymann G (1996). Body composition and energy expenditure in thyroidectomized patients during short-term hypothyroidism and thyrotropin-suppressive thyroxine therapy. Eur. J. Endocrinol..

[40] Little AG (2016). A review of the peripheral levels of regulation by thyroid hormone. J. Comp. Physiol. B.

[41] Brent GA (2012). Mechanisms of thyroid hormone action. J. Clin. Invest..

[42] Haluzik M, Nedvidkova J, Bartak V (2003). Effects of hypo- and hyperthyroidism on noradrenergic activity and glycerol concentrations in human subcutaneous abdominal adipose tissue assessed with microdialysis. J. Clin. Endocrinol. Metab..

[43] Gnocchi D, Steffensen KR, Bruscalupi G (2016). Emerging role of thyroid hormone metabolites. Acta Physiol..

[44] Visser M, Deurenberg P, van Staveren WA (1995). Resting metabolic rate and diet-induced thermogenesis in young and elderly subjects: relationship with body composition, fat distribution, and physical activity level. Am. J. Clin. Nutr..

[45] Dabbech M, Aubert R, Apfelbaum M (1994). Reproducible expenditure measurement of postprandial in young healthy males. Am. J. Clin. Nutr..

[46] Wahrenberg H, Wennlund A, Arner P (1994). Adrenergic regulation of lipolysis in fat cells from hyperthyroid and hypothyroid patients. J. Clin. Endocrinol. Metab..

[47] Penev P, Spiegel K, Marcinkowski T (2005). Impact of carbohydrate-rich meals on plasma epinephrine levels: dysregulation with aging. J. Clin. Endocrinol. Metab..

[48] Oomen JM, Waijers PMCM, van Rossum C (2005). Influence of ß2-adrenoceptor gene polymorphisms on diet-induced thermogenesis. Br. J. Nutr..

